# Infective Endocarditis Presenting as Rhabdomyolysis and Muscle Abscess: A Case Report

**DOI:** 10.7759/cureus.49682

**Published:** 2023-11-29

**Authors:** Naseem Ambra, Azeez Palol, Muhammed J Moidy, Ahmad Kordi, Salah Almughalles, Abdulqadir J Nashwan

**Affiliations:** 1 Internal Medicine, Hamad Medical Corporation, Doha, QAT; 2 Medical Imaging, Hamad Medical Corporation, Doha, QAT; 3 Nursing, Hamad Medical Corporation, Doha, QAT

**Keywords:** reactive arthritis, streptococcus dysgalactiae, acute kidney injury, infective endocarditis, rhabdomyolysis

## Abstract

Rhabdomyolysis is characterized by the degradation of skeletal muscle tissue, which releases cellular contents into circulation. This condition commonly stems from various factors, including trauma, overexertion, muscular hypoxia, infections, metabolic and electrolyte imbalances, certain medications, toxins, and genetic abnormalities. Despite this, instances of rhabdomyolysis precipitated by bacteremia of infective endocarditis remain exceedingly rare. This report describes an unusual case wherein infective endocarditis manifested as rhabdomyolysis, accompanied by a muscular abscess and acute renal failure. The patient's condition was successfully managed through hydration and targeted antibiotic therapy, leading to a favorable recovery. The case underscores the importance of vigilance for extracardiac symptoms and signs of infective endocarditis, such as rhabdomyolysis and muscular abscesses. Of particular note in this case was the discovery of an atypical causal bacterium, *Streptococcus dysgalactiae*, in the setting of infective endocarditis. This case highlights the broad range of potential manifestations and causal factors associated with this serious cardiac condition.

## Introduction

Infective endocarditis (IE) is a potentially fatal illness, with an in-hospital mortality as high as 18% [1]. Staphylococci, streptococci, and enterococci are the three most frequent causes of IE worldwide [2]. Streptococci from groups A and B cause most IE, while groups C and G are responsible for less than 2% of cases [3]. *Streptococcus dysgalactiae* is a streptococcus of the Lancefield group C or G. This bacterium primarily causes skin and soft-tissue infections. However, invasive infections can also happen [3]. Sepsis is frequently linked to rhabdomyolysis, and gram-positive bacterial infections are said to be the most common cause of sepsis-induced rhabdomyolysis [4]. We report a case of IE presenting as rhabdomyolysis and muscle abscess caused by *S. dysgalactiae* bacteremia. 

## Case presentation

A 35-year-old Asian, healthy male, who worked as a manual laborer, was admitted to our hospital with complaints of tiredness and generalized body pain for the past five days. The left upper limb and lower limbs were more painful. He did not go to work or engage in strenuous physical activity for one week before the presentation. He denied having fever, cough, or chest pain. There was no history of smoking or alcohol consumption, intravenous drug misuse, chronic medication use, or recent travel. 

At the time of admission, the patient's vitals were: temperature 37.2 C, blood pressure 110/57 mmHg, respiratory rate 18 minutes per minute, and oxygen saturation in room air 97%. There was mild generalized edema and tenderness in the muscles. The left upper limb was swollen and significantly tender. Cardiovascular exams revealed normal heart sounds and no added sounds or murmur. Other system examinations were unremarkable. Table 1 presents a detailed summary of laboratory results upon patient admission. The listed parameters include complete blood count, renal function tests, electrolytes, liver function tests, inflammatory markers, immunological tests, and coagulation profile, providing a comprehensive overview of the patient's physiological status.

**Table 1:** Laboratory results on admission WBC: White Blood Cell Count; RBC: Red Blood Cell Count; HGB: Hemoglobin; HCT: Hematocrit; MCV: Mean Corpuscular Volume; MCH: Mean Corpuscular Hemoglobin; MCHC: Mean Corpuscular Hemoglobin Concentration; RDW-CV: Red Cell Distribution Width-Coefficient of Variation; BUN: Blood Urea Nitrogen; C3: Complement Component 3; C4: Complement Component 4; ANCA: Anti-Neutrophil Cytoplasmic Antibodies; GBM: Glomerular Basement Membrane; int: Interpretation; ALP: Alkaline Phosphatase; ALT: Alanine Aminotransferase; AST: Aspartate Aminotransferase; INR: International Normalized Ratio; PT: Prothrombin Time; APTT: Activated Partial Thromboplastin Time; LDH: Lactate Dehydrogenase; TSH: Thyroid-Stimulating Hormone; HbA1C: hemoglobin A1c

Parameter	Patient's Value	Reference Range
WBC (x10^3^/uL)	6.6	4-10
RBC (x10^6^/uL).	4.9	4.5-5.5
HGB (gm/dL)	13.2	13-17
HCT (%)	36.8	40-50
MCV (FL)	74.6	83-101
MCH (PG)	26.8	27-32
MCHC (gm/dL)	35.9	31.5 – 34.5
RDW-CV (%)	13.4	11.6- 14.5
Platelets (x10^3^/uL)	60	150-400
Absolute neutrophil count auto (x10^3^/ul)	5.4	2-7
BUN (mmol/L)	32.8	2.5-7.8
Creatinine (umol/L)	612	62 - 106
Sodium (mmol/L)	128	133-146
Potassium (mmol/L)	4.5	3.5-5.3
Chloride (mmol/L)	93	95-108
Bicarbonate (mmol/L)	15	22-29
Adjusted Ca++ (mmol/L )	2.41	2.20-2.60
Phosphorous (mmol/L )	2.05	0.80-1.50
Magnesium (mmol/L)	0.94	0.70-1.00
HbA1C (%)	5.9	>6.5 Diabetes
Bilirubin TOTAL (umol/L)	21	0-21
Bilirubin DIRECT (umol/L)	12	0 - 5
Total protein (gm/L)	66	60-80
Albumin (gm/L)	24	35-50
Uric acid (umol/L)	702	200-403
Creatine kinase (U/L)	1269	39-308
Myoglobin (ng/mL)	4284	28-72
C-reactive protien (mg/L)	340	0.0 – 5.0
C3 (gm/L)	0.97	0.9 – 1.8
C4 (gm/L)	0.18	0.1 – 0.4
ANCA	Negative	
Anti-GBM antibody (U/mL)	<1.9	
Anti-GBM antibody (int)	Negative	
ALP (U/L)	158	40-129
ALT (U/L)	40	0-41
AST (U/L)	95	0-40
INR	1	Critical high >4.9
PT (seconds)	12.2	9.4-12.5
APTT (seconds)	37.2	25.1 – 36.5
D-Dimer (mg/L )	4.03	0.00 – 0.49
LDH (U/L)	289	135-225
Lactic acid (mmol/L)	0.9	0.5-2.2
Procalcitonin (ng/ml)	4.30	<0.5
Ferritin (ug/L)	811	48-420
TSH (mIU/L)	0.85	0.30-4.20

The blood gas analysis detected high anion gap metabolic acidosis (Table 2). Screening for HIV, hepatitis B, hepatitis C, respiratory viruses, and coronavirus disease 2019 (COVID-19) were negative. Urine analysis showed microscopic hematuria. A provisional diagnosis of rhabdomyolysis with acute kidney injury was made, and intravenous hydration was initiated. Nephrology services were consulted for the management of acute renal failure.

**Table 2:** Venous blood gases on admission pH Ven: Venous pH; PCO2 Ven: Venous Partial Pressure of Carbon Dioxide; PO2 Ven: Venous Partial Pressure of Oxygen; Ca ++: Calcium; SPO2: Oxygen Saturation; O2 Hb: Oxyhemoglobin; CO Hb: Carboxyhemoglobin; Met Hb: Methemoglobin; HCO3: Bicarbonate; T CO2: Total Carbon Dioxide

Venous Blood Gas Report	Patient’s Value	Reference Range
pH Ven	7.291	7.32-7.42
PCO2 Ven	36 mmHg	41-51 mmHg
PO2 Ven	43 mmHg	25-40 mmHg
Sodium	128 mmol/L	135-145 mmol/L
Ptassium	4.3 mmol/L	3.5-5 mmol/L
Chloride	97 mmol/L	96-110 mmol/L
Ca ++	1.09 mmol/L	1.18-1.32 mmol/L
Glucose	5.4 mmol/L	3.3-5.5 mmol/L
Lactate	1.60 mmol/L	0.50 -2.20 mmol/L
SpO2	74.5 %	95-99 %
O2 Hb	72.9 %	94-98 %
CO Hb	1.2 %	0.5-1.5 %
Met Hb	1.0 %	0.0-1.5 %
HCO3	17.1 mmol/L	23-29 mmol/L
T CO2	18.2 mmol/L	23-27 mmol/L
Base excess	-8.6 mmol/L	-2.0 – 2.0 mmol/L

**Figure 1:**
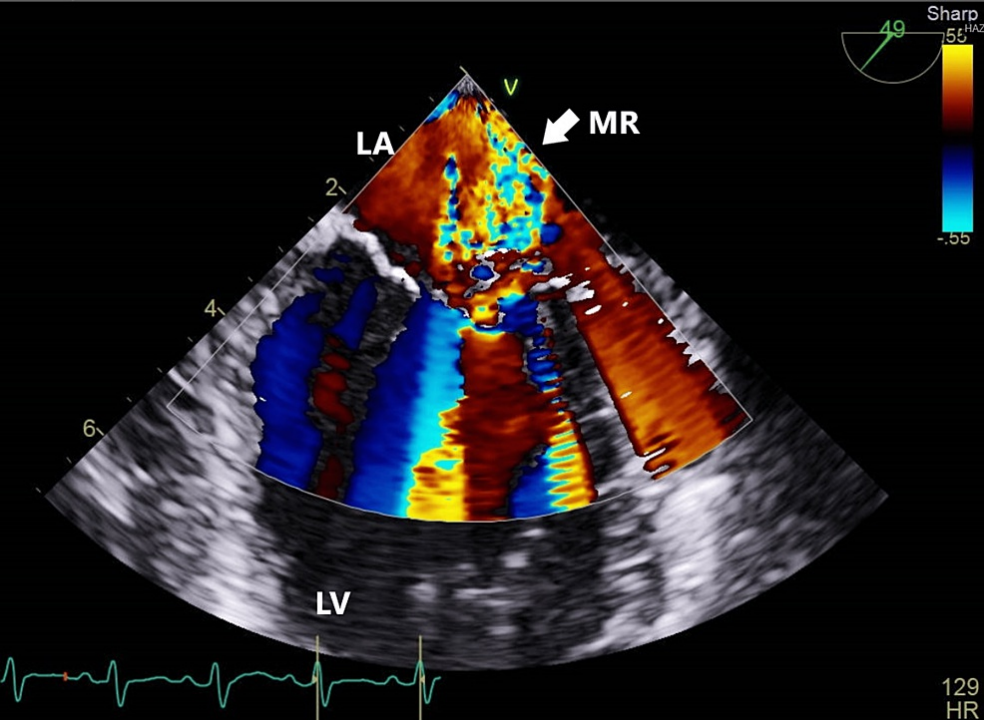
Mitral regurgitation LA: Left Atrium; LV: Left Ventricle; MR: Mitral Regurgitation

ECG showed sinus tachycardia. Chest radiography was normal. Abdominal ultrasound revealed normal-sized kidneys with increased echogenicity, and ultrasound Doppler of the left upper limb was normal. On the third day after admission, an echocardiogram revealed severe mitral regurgitation with a possible partial flail leaflet (Figure 1).

**Figure 2:**
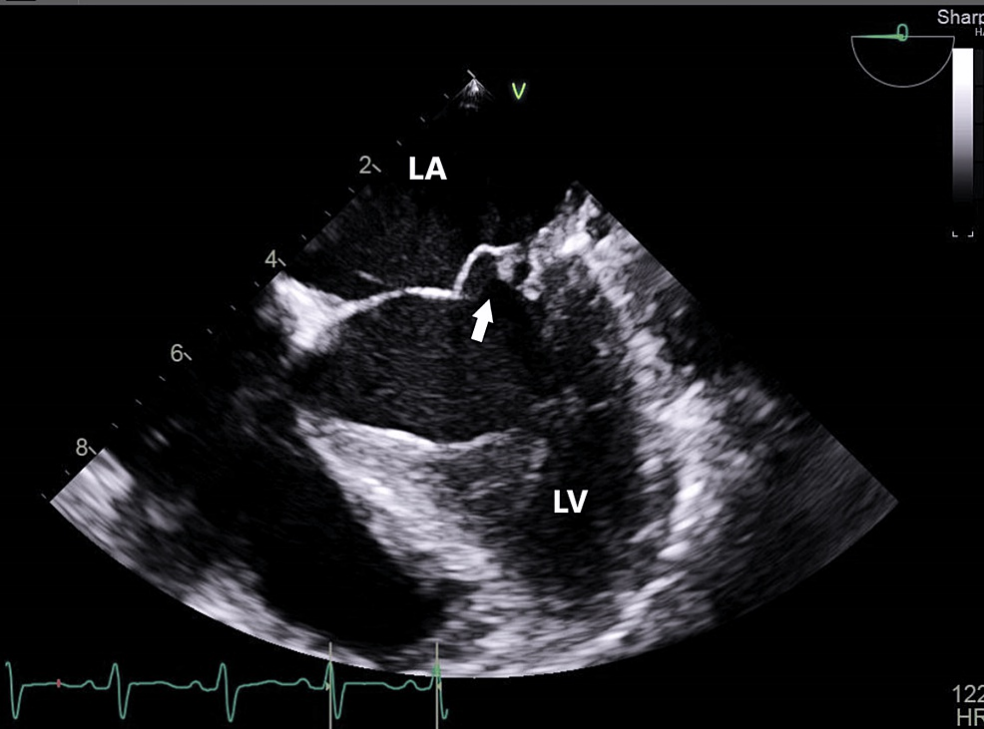
AML vegetations AML: Anterior Mitral Leaflet; LA: Left Atrium; LV: Left Ventricle

**Figure 3:**
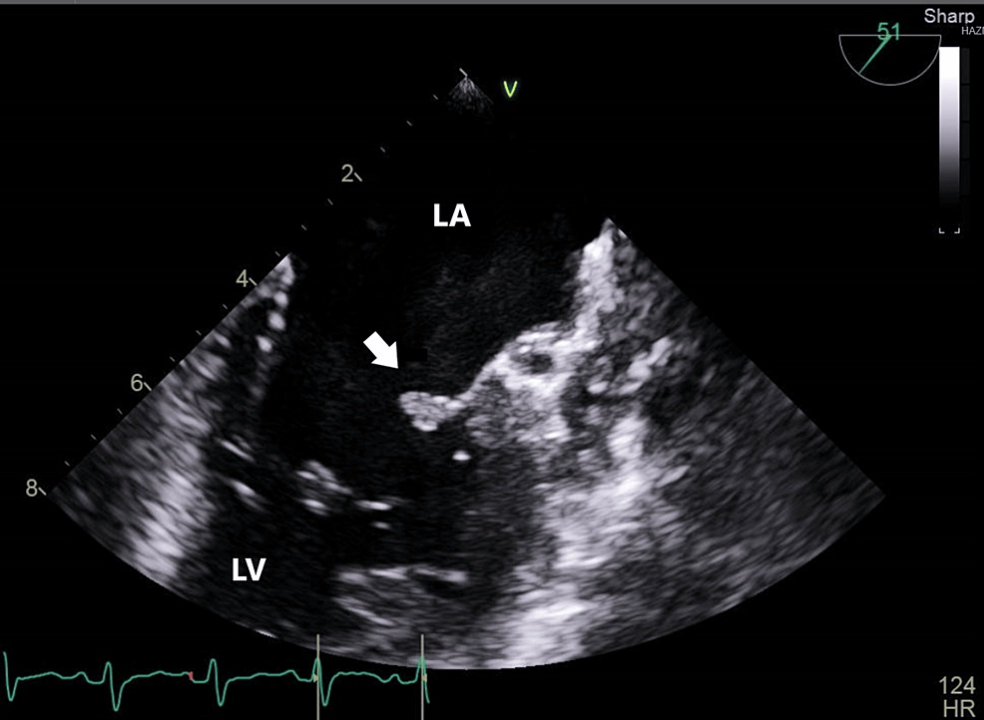
PML vegetations PML: Posterior Mitral Leaflet; LA: Left Atrium; LV: Left Ventricle

A subsequent transesophageal echo (TEE) revealed moderate to severe mitral regurgitation and medium to large-size mobile echogenic mass (1.1 x 0.7 cm) attached to the anterior and posterior leaflets of the mitral valve (Figures 2, 3). There is severe prolapse of the anterior and posterior leaflet and flail motion of the anterior mitral leaflet, possibly secondary chordae tendineae rupture. Other valves were normal. There was no root abscess or destruction of the valve structure. The cardiology and cardiothoracic surgery teams were involved in the patient's care. It was decided that medical management should proceed with valve replacement surgery at a later stage after the acute infection is cured.

**Figure 4:**
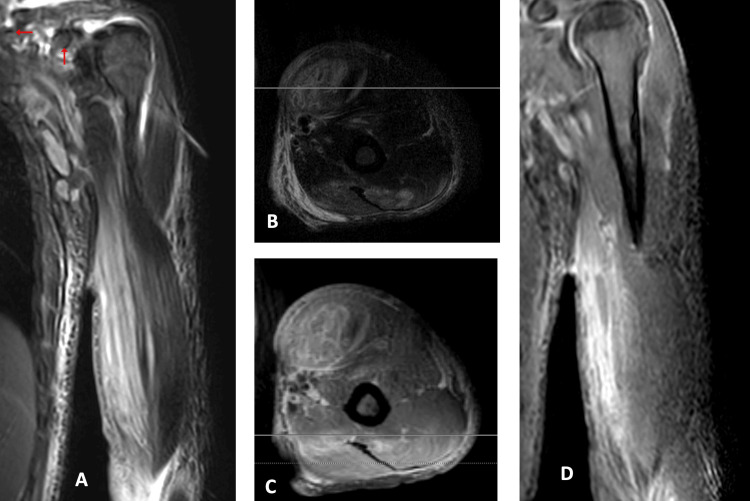
Biceps muscle abscess The medial head of the biceps muscle is diffusely enlarged and shows abnormal fluid signal (Coronal image (A) and Axial image (B) of T2 fat Sat) that shows faint enhancement with multilocular abnormal heterogeneous fluid signal lesions of ring enhancement; small abscesses (Axial T1 post-contrast image (C)) as well as diffuse faint surrounding muscle enhancement (Coronal T1 post-contrast image (D)).

**Figure 5:**
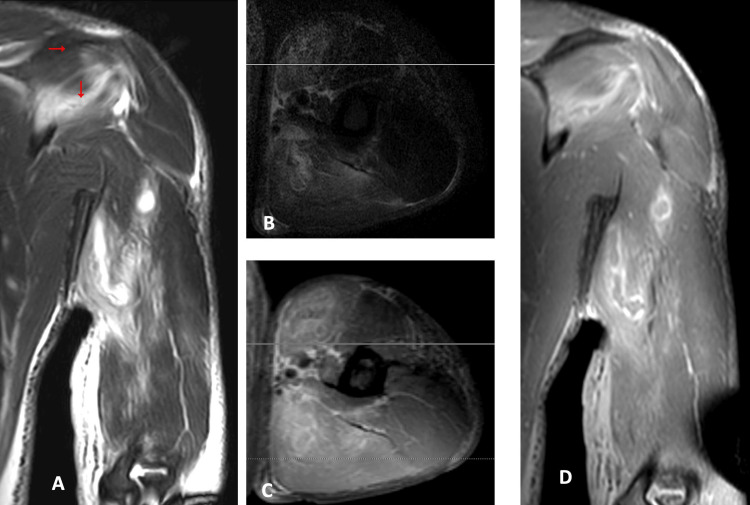
Triceps muscle abscess The medial head of the triceps muscle is diffusely enlarged and shows abnormal fluid signal (Coronal image (A) and axial image (B) of T2 fat Sat) that shows faint enhancement with multilocular abnormal heterogeneous fluid signal lesions of ring enhancement; small abscesses (Axial image (C) and coronal image (D) of T1 post-contrast).

**Figure 6:**
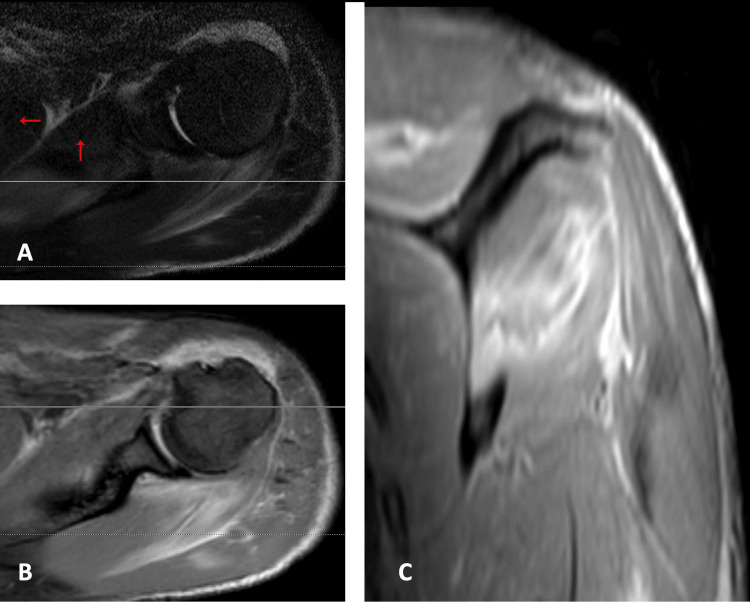
Rotator cuff edema The rotator cuff muscles, especially the supraspinatus and infraspinatus, are oedematous and demonstrate hyperintense signal in the fluid-sensitive sequences (Axial T2 Fat Sat image (A)) and show diffuse heterogenous contrast enhancement on post-contrast T1 sequences (Axial image (B) and coronal image (C)).

On the third day of admission, a blood culture was reported, and it revealed the presence of *S. dysgalactiae*, sensitive to ceftriaxone. MRI of the left upper limb showed multiple small abscesses and features suggestive of necrotizing fasciitis (Figures 4-6). Given that the abscess was small, and the patient's clinical condition was improving with antibiotics, no surgical procedures were performed.

A repeat blood culture reported negative results five days after starting antibiotics. Blood urea and serum creatinine decreased daily and returned to normal on day 10 of hospitalization. Urine output was normal throughout the course (nonoliguric acute kidney injury).

During the third week following admission, he started experiencing pain and swelling on his left wrist, which gradually got worse. The results of the ultrasound Doppler and MRI of the left wrist suggested arthritis. The rheumatology and orthopedic team were involved. Surgery was performed on the left wrist on day 29 of admission. Left wrist joint arthrotomy with tissue biopsy and culture was done. The histopathology results indicated reactive arthritis without any pus being drained.

**Figure 7:**
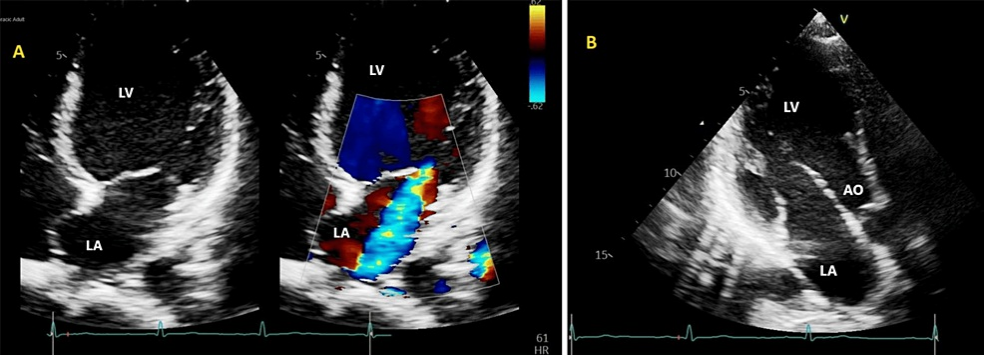
Follow-up transthoracic echocardiogram images (A) Mitral regurgitation in the color Doppler view; (B) View of the left atrium (LA), left ventricle (LV), and aorta (AO).

**Figure 8:**
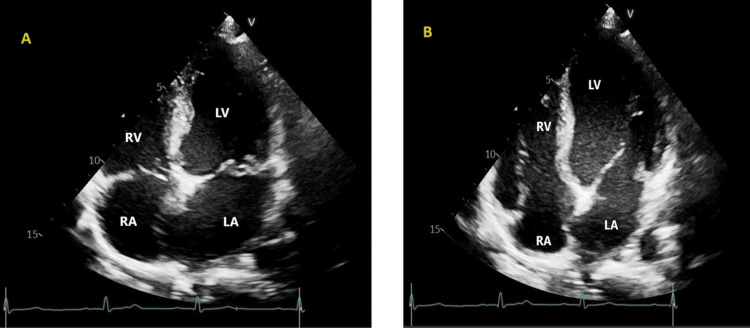
Follow-up transthoracic echocardiogram images (A) Systolic phase, when the heart muscle contracts; (B) Diastolic phase, when the heart muscle relaxes. LV: Left Ventricle; LA: Left Atrium; RV: Right Ventricle; RA: Right Atrium

Transthoracic echocardiography (TTE) was done on day 12 of admission and repeated on day 30 of admission (Figure 7, 8), which revealed mildly dilated left atrium and left ventricles, normal left ventricular ejection fraction (LVEF), degenerative mitral valve disease (mild mitral valve prolapse (MVP)), and moderate mitral regurgitation.

Then, 35 days after being hospitalized, he was discharged. IV antibiotic was given for an additional 10 days after being discharged. On examination at the clinic three weeks following discharge, he was symptom-free except for a slight left wrist pain. Functional ability and laboratory results were normal. He was scheduled to have valve replacement surgery, but he chose to have it in his home country instead (Table 3).

**Table 3:** Timeline of events

Time	Events
Day 0 [day of admission]	Diagnosis of rhabdomyolysis and acute renal failure
Day 3	1- Transthoracic echocardiogram revealed MR
	2- Blood culture reported as *Streptococcus dysglactiae*
	3- Ultrasound of kidney and ultrasound Doppler of upper limb
Day 5	Transesophageal echocardiogram revealed infective endocarditis
Day 6	MRI of the left upper limb revealed a micro abscess and necrotizing fasciitis
Day 8	Repeat blood culture negative
Day 10	Renal parameters normal
Day 12	Repeat transthoracic echocardiogram
Day 17	Ultrasound Doppler upper limb
Day 20	Repeat MRI of upper limb
Day 21	Ultrasound hand and wrist showed synovitis
Day 27	MRI of the left upper limb showed septic arthritis at the left wrist joint
Day 29	Arthrotomy left wrist
Day 30	Repeat transthoracic echocardiogram
Day 35	Discharge home
Day 45	Review in clinic

## Discussion

Infection of the endocardial surface of the heart is known as IE. It can present as an acute, rapidly progressing infection or as a subacute or chronic condition with low-grade fever and vague symptoms. Up to 96% of people with IE experience fever as their primary symptom [5]. Chills, anorexia, and weight loss are frequently linked to it. Regardless of whether a fever is present, patients with IE often have ongoing bacteremia. IE can also cause malaise, headaches, myalgias, arthralgias, night sweats, stomach pain, and dyspnea. Rheumatological symptoms such as back pain, myalgias, arthralgias, and arthritis might also be a symptom of IE [6].

This patient was presented with rhabdomyolysis and acute renal failure. The diagnosis of rhabdomyolysis was made based on the presence of severe myalgia and a rise in creatine phosphokinase and myoglobin to levels that were many times over the usual upper limit. A positive blood culture led to further evaluations, and IE was diagnosed.

Only a few cases of rhabdomyolysis associated with IE are reported. One similar case was reported by Mikaberidz et al. describing IE complicated by rhabdomyolysis and sudden permanent hearing loss. Causative organism was *Pasteurella multocida* [7]. Another case of IE with intercoastal muscle abscess caused by *Staphylococcus aureus* was reported by Nakayama et al. [8].

Intravenous drug users (IVDU) are typically associated with the development of subcutaneous soft tissue infections, which can lead to serious conditions like rhabdomyolysis, sepsis, and, in rare cases, IE [9,10]. However, we encountered a unique case involving a non-IVDU individual. This case is intriguing as the muscular injury observed could have stemmed from sepsis associated with IE since there were no evident causes for rhabdomyolysis, such as trauma, seizures, or medication side effects in this patient [11]. Interestingly, bacterial sepsis is implicated in approximately 5% of rhabdomyolysis cases [12]. Research indicates that rhabdomyolysis is more commonly linked to gram-positive bacteremia than gram-negative sepsis [11,12]. The proposed mechanisms by which sepsis might induce rhabdomyolysis include direct invasion of muscle tissue by pathogens, toxin production, cytokine-mediated muscle cell damage, and ischemia due to shock [13-16].

Deep abscesses are known to develop IE caused by organisms with pyogenic potential [6]. *Staphylococcus pyogenes* is the bacterium most associated with deep infections. In our patient, *S. dysgalactiae* was the causative organism, which has already been reported to produce IE [17]. In the reported cases of IE due to group G *Streptococcus*, the left side of the heart was primarily affected. In around 50% of cases, endocarditis developed on a normal valve. IE due to *S. dysgalactiae* is an aggressive condition, and most patients experience cardiac and embolic complications, and the mortality rate is significant (36%) [18,19]. The high rate of systemic emboli may be explained by the large size and friability of the vegetations [20-22]. *S. dysgalactiae* isolates remain always sensitive to penicillin and other beta-lactam medications. [18]. The current patient was treated with ceftriaxone, and a repeat blood culture was reported negative after five days of antibiotic therapy. IE caused by *S. dysgalactiae *may also require heart surgery due to its rapidly destructive nature, and 40% of the patients in the published series underwent heart surgery [23].

Post-streptococcal reactive arthritis occurs around 10 days after an infection [24]. Reactive arthritis is sterile synovitis that develops in a genetically predisposed person because of an infection localized in a different organ or system [24]. Treatment consists of non-steroidal anti-inflammatory drugs (NSAIDs) and steroids.

## Conclusions

This case report highlights the critical importance of acknowledging the broader clinical spectrum of IE, which extends beyond the classic cardiac manifestations. It brings to light the less common but consequential extra-cardiac complications, particularly the occurrence of rhabdomyolysis and muscular abscesses, which are not typically associated with IE. These atypical presentations can challenge the diagnostic process and potentially delay appropriate treatment. Moreover, the identification of *S. dysgalactiae* as the causative agent in this instance is notable for its rarity in clinical settings, as this bacterium is not commonly implicated in IE. This emphasizes the need for a high index of suspicion and comprehensive diagnostic approaches, including blood cultures and imaging, to detect and identify unusual pathogens in patients presenting with non-specific symptoms that could be attributed to IE.
